# Multimodal learning of clinically accessible tests to aid diagnosis of neurodegenerative disorders: a scoping review

**DOI:** 10.1007/s13755-023-00231-0

**Published:** 2023-07-22

**Authors:** Guan Huang, Renjie Li, Quan Bai, Jane Alty

**Affiliations:** 1 School of ICT, University of Tasmania, Sandy Bay, TAS 7005 Australia; 2 Wicking Dementia Research and Education Centre, University of Tasmania, Hobart, TAS 7000 Australia; 3 School of Medicine, University of Tasmania, Hobart, TAS 7000 Australia; 4 Neurology Department, Royal Hobart Hospital, Hobart, 7000 Australia

**Keywords:** Artificial intelligence, Alzheimer’s disease, Parkinson’s disease, Multimodal learning, Diagnosis, Age-related diseases, Pre-clinical, Multiple biomarkers

## Abstract

With ageing populations around the world, there is a rapid rise in the number of people with Alzheimer’s disease (AD) and Parkinson’s disease (PD), the two most common types of neurodegenerative disorders. There is an urgent need to find new ways of aiding early diagnosis of these conditions. Multimodal learning of clinically accessible data is a relatively new approach that holds great potential to support early precise diagnosis. This scoping review follows the PRSIMA guidelines and we analysed 46 papers, comprising 11,750 participants, 3569 with AD, 978 with PD, and 2482 healthy controls; the recency of this topic was highlighted by nearly all papers being published in the last 5 years. It highlights the effectiveness of combining different types of data, such as brain scans, cognitive scores, speech and language, gait, hand and eye movements, and genetic assessments for the early detection of AD and PD. The review also outlines the AI methods and the model used in each study, which includes feature extraction, feature selection, feature fusion, and using multi-source discriminative features for classification. The review identifies knowledge gaps around the need to validate findings and address limitations such as small sample sizes. Applying multimodal learning of clinically accessible tests holds strong potential to aid the development of low-cost, reliable, and non-invasive methods for early detection of AD and PD.

## Introduction

Neurodegenerative disorders are conditions that predominantly affect cells in the brain called neurons. When neurons ‘degenerate’ (become damaged or die), there is a loss of activity and, depending on which part of the brain is affected, there are progressive problems with cognitive and movement function. The most common types of neurodegenerative disorders are Alzheimer’s disease (AD) and Parkinson’s disease (PD), which are predominantly cognitive and movement disorders respectively, but others include motor neuron disease (MND), Lewy body dementia (LBD) and frontotemporal dementia (FTD). For the most part, it remains unclear why some people develop neurodegenerative disorders and others do not, but age is the biggest risk factor for nearly all cases. There are considerably higher rates of these disorders in older adults; for example, in the population of adults aged 60–70 about 1 in 10 have AD [1], and 1 in 100 have PD [2]; in the population of adults aged over 85, these figures are almost 1 in 3, and 4 in 100, for AD and PD respectively. As populations are ageing around the world, the prevalence of neurodegenerative disorders is thus rising, and there are already about 50 million people with AD and 10 million with PD [3, 4]. There is an urgent and growing need to find new ways of aiding early diagnosis of these conditions—to support better care, earlier recruitment to drug trials and new drug development.

AD is the most common cause of dementia—a progressive degenerative disorder of the brain that causes impaired cognition and functioning. It is a major health and social issue for all countries around the world and has been described by the Lancet Commission as *the greatest global challenge for health and social care in the twenty-first century* [5, 6]. Although AD manifests primarily as a cognitive disorder, there are also abnormalities of movement, such as slowed gait, reduced dexterity and speech and swallowing problems. The assessment and diagnosis of AD, and other types of dementia, typically involves a comprehensive clinical evaluation comprising gathering information about symptoms from the person, their family and/or caregivers, obtaining a detailed personal and family medical history, and a physical neurological examination. Cognitive assessments are required to evaluate various domains such as memory, language, perceptual skills, attention, constructive abilities, orientation, problem solving, and functional abilities [7]. Brain imaging, typically with structural magnetic resonance imaging (MRI) brain scans, and blood tests are also undertaken to look for evidence of degeneration (seen as atrophy, or ‘thinning’ of certain areas of the brain) and to rule out reversible causes of cognitive decline, such as vitamin deficiencies or other pathology [8].

**Fig. 1:**
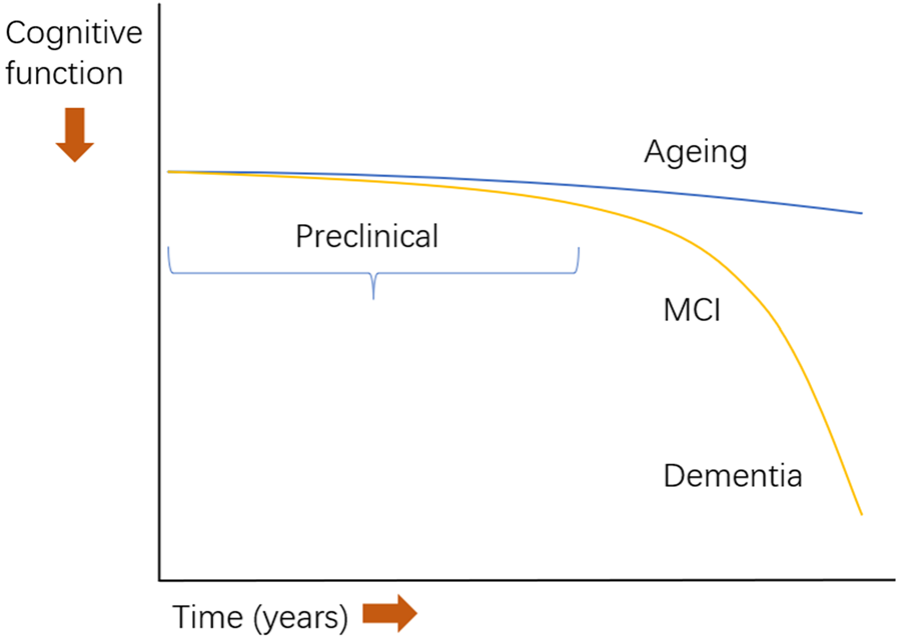
Model of the cognitive function decline trajectory of Alzheimer’s disease (AD) vs normal ageing. The stage of preclinical AD precedes with mild cognitive impairment (MCI), graph adapted from [10]

The pathology of AD is characterized by a gradual build-up of abnormal amyloid and tau proteins in the brain followed by neurodegeneration. This gradual accumulation of proteins occurs over a 10–15 year period before any classical cognitive symptoms of memory impairment emerge. There is thus a preclinical AD stage where pathology is present but there are no significant cognitive symptoms or decline in cognitive function. This critical stage, when interventions (such as drug trials) have the best chance of being effective, is typically only identified in research settings using specialist and expensive tests [9]. Figure 1 demonstrates how cognitive function declines with AD and normal ageing. Most people present clinically when they are in the Mild Cognitive Impairment (MCI) stage characterized by minor reductions in performance on cognitive tests without any functional impact on everyday activities [10]. As cognitive function gradually declines in AD, it impairs the ability to undertake everyday activities and at this advanced stage of pathology, is termed ‘dementia’.

Over the last decade, many new biomarkers have been developed to help detect AD pathology across the continuum, including blood-based biomarkers [11], cerebrospinal fluid (CSF) tests [12] and positron emission tomography (PET) scans [13]. However, these tests are invasive, costly and largely clinically inaccessible. There is thus increasing interest in how new techniques may be applied to data from tests that are already clinically accessible to aid diagnosis; these tests include measures of gait [14], speech [15], handwriting [16] and MRI [17]. The challenge will be developing automated objective methods to analyse this data, ideally in combination, to form accurate efficient tools that can be used in standard clinics.

PD is the second most common neurodegenerative disorder and is characterized by the build-up of Lewy bodies (comprising abnormal proteins such as Alpha-synuclein and ubiquitin) in the brain and progressive loss of dopamine containing neurons. It typically presents with impaired motor (movement) function manifesting as tremor, muscle rigidity and slowness of movement [18]. Other motor signs include quiet speech, reduced facial expression and small handwriting. However, non-motor symptoms, such as cognitive impairment and dementia, are also very common in PD. Studies suggest that up to 80% of people with PD will develop some form of cognitive impairment, with up to 50% eventually developing dementia [19]. Other neurological conditions, and even drug side effects, may mimic PD. The assessment and diagnosis of PD thus typically involves a comprehensive clinical evaluation comprising a detailed medical and drug history and a physical neurological examination by a specialist. MRI brain scans are usually normal in PD, or just show a mild degree of generalized atrophy, that overlaps with changes seen in normal ageing. The diagnosis of PD largely relies on clinician interpretation of clinical signs but about 20% of diagnoses are inaccurate, especially in the early stages [20].

Thus, both PD and AD are neurodegenerative disorders that manifest with a combination of progressive cognitive and motor abnormalities, and both rely heavily on detailed clinical assessments for diagnosis. With the rapidly rising prevalence of these conditions, there is a growing need for clinically-accessible tools that would aid an early diagnosis—to improve effective management, increase access to clinical trials and support new drug development.

Over recent years, Artificial intelligence (AI) has shown promising results in aiding the early detection of dementia [21], such as extracting more features from standard cognitive tests and improving the discriminatory sensitivity of MRI scans [22]. Multimodal AI is a new AI paradigm that seeks to create models for integrating and processing information from multiple modalities [23]. This cutting-edge method offers new approaches to aiding detection of neurodegenerative disorders as it allows different modal contents (cognitive score data, text data, image data, video data, audio data, etc.) that can be analyzed together [24]. This is a significant development for detection of AD and PD, as it means that all the data that is already collected in standard clinical assessments can be analyzed together to form potentially much more accurate models; for example the text data from clinical history, the numerical data from cognitive tests, the image data from MRI scans, the audio data from voice recordings etc. It is a highly attractive approach for healthcare as it does not add any additional costs or time, uses data that is already clinically accessible, and holds potential for cost savings—though identification of the most discriminatory tests as well as though earlier diagnosis. Multimodal AI significantly advances previous AI methods of ‘single modal’ analysis where a single type of diagnostic tool, such as a cognitive test or a brain scan, are analyzed to automate a prediction of likelihood for AD or PD.

There are no previous reviews specifically examining the accuracy of multimodal AI techniques applied to clinically accessible data to aid early detection of AD or PD. This is an important knowledge gap to address as it will help inform which data sets and techniques show most promise for further development as clinical tools. In this study, our objective was to summarise the evidence of how multimodal analysis of clinically accessible data aids early detection of AD and PD.

This paper is organized as follows: Sect. 2 introduces the method used to construct the scoping review, Sect. 3 presents the results and the characteristics of the evidence, Sect. 4 provides a narrative synthesis of relevant findings and Sect. 5 discusses the conclusions and future directions.

## Method

### Study design and research question

This scoping review followed the guidelines of the Preferred Reporting Items for Systematic Reviews and Meta-analyses(PRISMA) [25]. The focus of the scoping review was to summarise the evidence on multimodal features in the two most common types of neurodegenerative disorder: AD and PD, to aid diagnosis and to identify research gaps. The research question was: *How does multimodal learning of clinically accessible data assist in the early diagnosis of AD and PD*?

### Eligibility criteria

Peer-reviewed original research papers, published in English between January 2012 and February 2023 were included if they comprised: (1) adults aged 18 or over with AD or PD, (2) focused on detecting/diagnosing/predicting AD or PD, and (3) multi modal data which was defined as at least 2 different modalities of data. Data modalities could include, but was not limited to, magnetic resonance imaging (MRI), computed tomography (CT), positron emission tomography (PET) image data, genetic data, clinical text data, video data, cognitive test (numerical, text, image) data, speech/audio data and movement (video, sensor, drawing, writing) data. Articles were excluded if they related to children or animals, only included a single data modality or were a review, systematic review, book chapter or single case report.

### Information sources and search strategies

We searched for eligible papers in two databases, PubMed and Scopus, with the following three major concepts: ‘neurodegenerative disorder’, ‘multimodal/multiple features’ and ‘classification/detection/diagnosis’. Free text terms and wild cards were used in the research, such as “degenerative”, “neurological”, “Alzheimer”, “Parkinson” for concept one, “multimodal”, “multichannel”, “multi-modal”, “multi features” for concept two, “fus*”, “detect*”, “diagnos*” for concept three. The full search query was: (TITLE-ABS-KEY (multimodal OR multichannel OR multi-modal OR multi AND features) AND TITLEABS-KEY (classification OR classify* OR categor*) AND TITLEABS-KEY (dementia OR degenerative OR neurological OR Alzheimer OR Parkinson) AND TITLE-ABS-KEY (fus* OR detect* OR diagnos*). The searches were limited to title, abstract and keywords.

### Selection of sources of evidence and data charting process

Two reviewers (GH plus JA or RL) independently screened each paper using the title and abstract according to the inclusion and exclusion criteria. Discrepancies were discussed between the reviewers until we made a consensus decision. A data extraction table was created by GH to collect data from each publication including year of publication, country, number of participants, neurodegenerative disorder type, data modalities collected, data analysis methods used and dataset size. The data extraction table was checked and confirmed by another reviewer (JA). Data were extracted by one reviewer (GH).

### Synthesis of results

The summary of the findings from each article was tabulated by GH and then checked by all the authors. All the authors have reviewed and summarised the findings and the gaps based on the available evidence through narrative synthesis.

## Results

### Selection of evidence

**Fig. 2:**
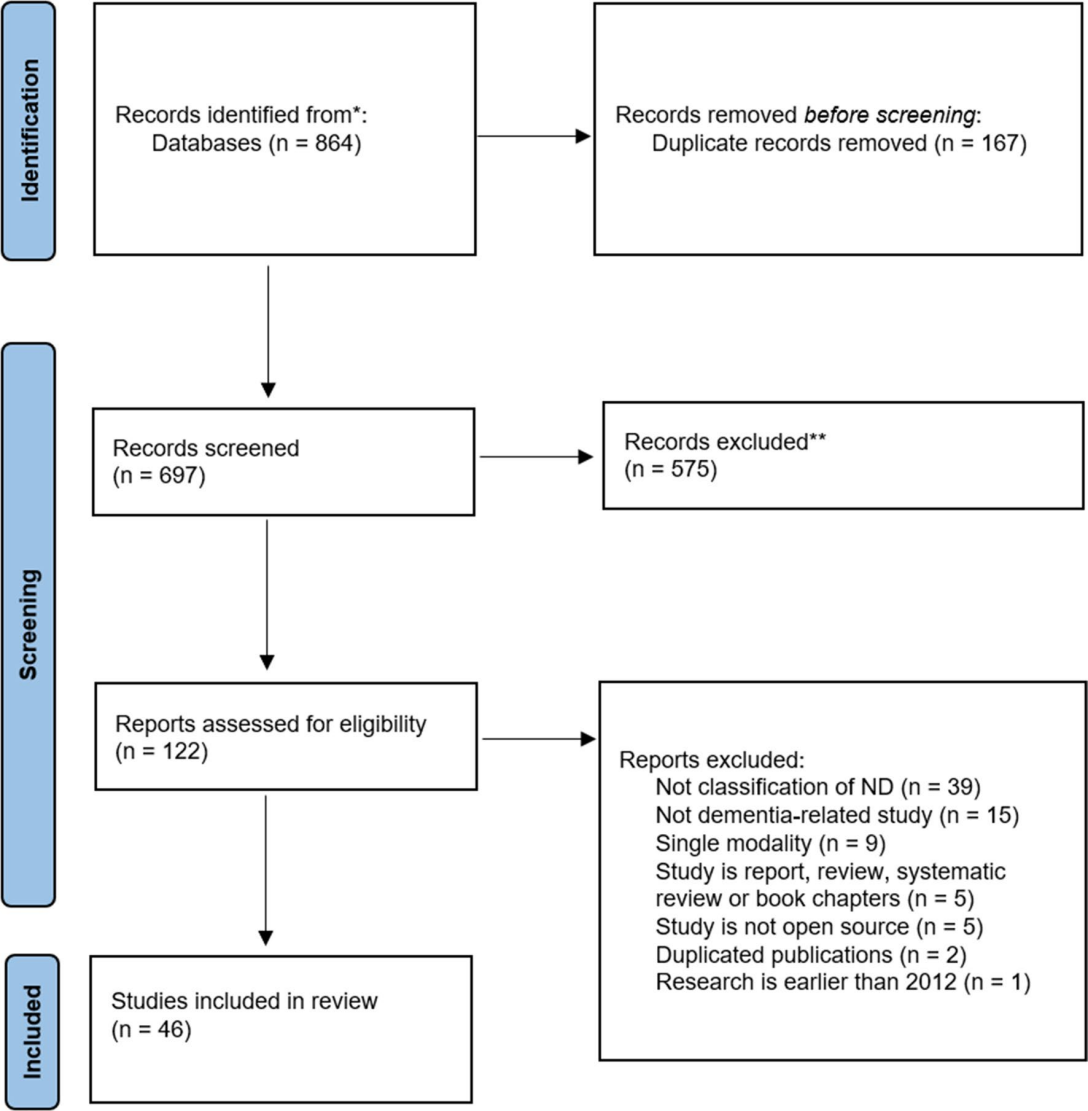
PRSIMA flow chart of the scoping review. This diagram shows the processing of scoping review, including identification, screening and the number of papers included in our study

The total number of electronic records yielded initially was 864 and 167 duplicated records were removed. The remaining 697 publications were screened and 575 were excluded as they did not meet the eligibility criteria. A total number of 46 articles were included in the review. The full search and selection process is shown in Fig. 2.

### Characteristics of the evidence

**Fig. 3:**
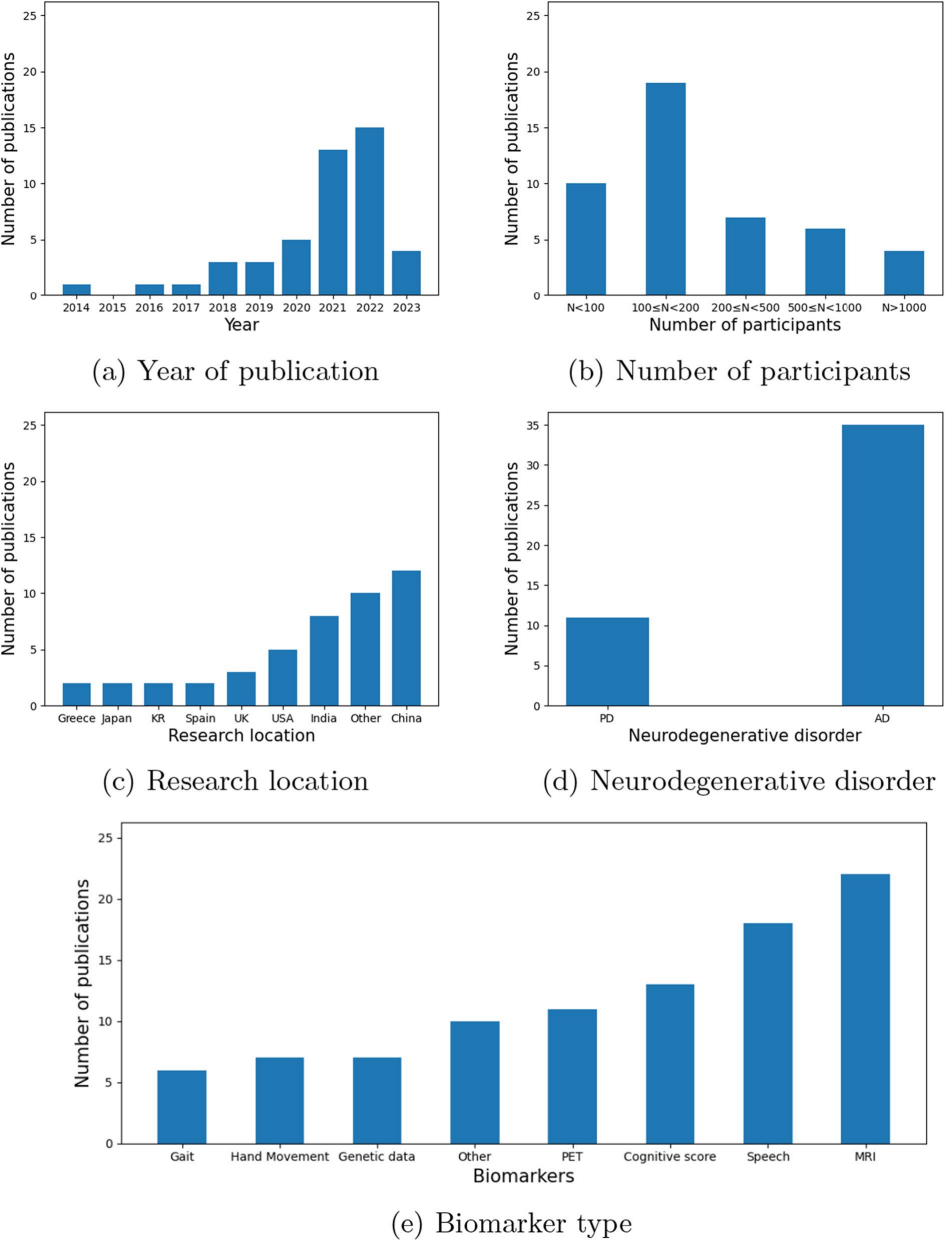
Meta-data from the review process

Table 1 summarises the characteristics of the 46 research papers; these comprised 11,750 participants in total: with 3569 with AD, 978 with PD and 2482 healthy controls. All the studies were cross-sectional, 39 focused on AD, 6 on PD, and all data was collected in clinical settings. The vast majority (40/46 studies) were published in the last 5 years. All studies contained a healthy control (HC) group and 15 AD studies also included an MCI group, comprising 4523 MCI participants in total. None of the studies included both AD and PD groups, but one AD study included another neurodegenerative disorder (LBD) group as well as healthy controls. Figure 3 summaries the meta-data of the included studies, with most publications from China (n = 12), India (n = 8) and the USA (n = 5), and most studies involving 100–200 participants. Imaging, speech and cognitive data were the most common modalities analysed with 24, 17 and 12 studies respectively including these types of data.

**Table 1:** Peer-reviewed primary research articles summary

First author	Country	Type	Biomarkers	Dataset^a,b^	Year	References
Suk	USA	AD	PET, MRI	ADNI (AD = 93, MCI = 204, HC = 101)	2014	[17]
Prashanth	India	PD	RBD^c^, EEG^c^, Olfactory function	PPMI (HC = 183, PD = 401)	2016	[26]
Shi	China	AD	PET, MRI	ADNI (AD = 51, MCI = 99, HC = 52)	2017	[27]
Pahuja	India	PD	MRI, Genetic data	PPMI (HC = 82, PD = 82)	2018	[28]
Garcia	USA	PD	Speech, Handwriting, Gait	Custom^d^ (HC = 41, PD = 49)	2018	[29]
Vasquez	Spain	PD	Speech, Handwriting, Gait	Custom (PD = 44, HC = 40)	2018	[30]
Pham	Singapore	PD	Hand movement, Speech	Custom (PD = 20, HC = 20)	2019	[31]
Noella	India	PD	Gait, MRI	PhysioNet (PD = 93, HC = 73)	2019	[32]
Zhou	China	AD	MRI, PET, Genetic data	ADNI (HC = 204, MCI = 362, AD = 171)	2019	[33]
Taleb	Lebanon	PD	Handwriting, Speech, RBD	PDMultiMC (PD = 21, HC = 21)	2020	[34]
Dachena	Italy	AD	MRI, Cognitive score	ADNI (HC = 36, AD = 33)	2020	[35]
Koo	South Korea	AD	Cognitive score, Speech	ADReSS (AD = 78, HC = 78)	2020	[36]
Martinc	Slovenia	AD	Cognitive score, Speech	ADReSS (AD = 78, HC = 78)	2020	[37]
Pompili	Portugal	AD	Text, Speech	ADReSS (AD = 78, HC = 78)	2020	[38]
Sánchez	Mexico	AD	Cognitive score, Genetic data	ADNI (HC = 36, MCI = 52, AD = 18)	2021	[39]
Song	China	AD	MRI, Genetic data, Cognitive score	TADPOLE (HC = 413, MCI = 865, AD = 337)	2021	[40]
Pandey	India	AD	Text, Speech	ADDreSSo (HC = 79, AD = 87)	2021	[41]
Arco	Spain	AD	MRI, PET, Cognitive score	ADNI (HC = 61, AD = 73)	2021	[42]
Dong	China	AD	MRI, PET, CSF^c^ data	ADNI (HC = 229, MCI = 405, AD = 188)	2021	[43]
Bi	China	PD	MRI, Genetic data	PPMI (HC = 49, PD = 55)	2021	[44]
Yang	China	PD	MRI, DTI^c^, Clinical information	PPMI (HC = 36, PD = 65)	2021	[45]
Nasreen	UK	AD	Text, Speech	Custom (AD = 15, HC = 15)	2021	[46]
Yamada	Japan	AD	Gait, Speech, Hand-drawing	Custom (AD = 26, MCI = 45, HC = 47)	2021	[47]
Nasreen	UK	AD	Speech, Medical history	CCC (AD = 15, HC = 15)	2021	[48]
Fukushima	Japan	AD	Speech, EEG	Custom (AD = 35, MCI = 18)	2021	[49]
Rohanian	UK	AD	Cognitive score, Speech	ADReSS (AD = 78, HC = 78)	2021	[50]
Jang	Canada	AD	Speech, RBD	Custom (HC = 83, MCI/AD = 79)	2021	[51]
Sheng	China	AD	MRI, Genetic data	ADNI (HC = 25, MCI = 50, AD = 25)	2022	[52]
Jiao	China	AD	MRI, PET	ADNI (HC = 79, MCI = 102, AD = 69)	2022	[53]
Ilias	Greece	AD	Cognitive score, Speech	ADReSS (AD = 78, HC = 78)	2022	[54]
Min	South Korea	AD	Gait, EEG	Custom (HC = 69, MCI = 151)	2022	[55]
Dolci	USA	AD	MRI, Genetic data	Custom (HC = 530, AD = 258)	2022	[56]
Hansen	Germany	AD	PET, CT scans	Custom (HC = 94, AD = 33)	2022	[57]
El-Sappagh	Egypt	AD	MRI, Cognitive score	ADNI (HC = 419, MCI = 613, AD = 339)	2022	[58]
Ying	China	AD	Text, Speech	ADReSSo (HC = 79, AD = 87)	2022	[59]
Moguilner	USA	AD	MRI, EEG, Cognitive score	Custom (HC = 152, AD = 76)	2022	[60]
Dwivedi	India	AD	MRI, PET	ADNI (HC = 100, MCI = 100, AD = 100)	2022	[61]
Habuza	UAE	AD	MRI, Cognitive score	ADNI (HC = 287, MCI = 646, AD = 369)	2022	[62]
Velazquez	USA	AD	DTI, Genetic data, Cognitive score	ADNI (HC = 335, MCI = 383, AD = 49)	2022	[63]
Shi	China	AD	MRI, PET	ADNI (HC = 52, MCI = 99, AD = 51)	2022	[64]
Safai	India	PD	MRI, DWI^c^	Custom (HC = 34, PD = 74)	2022	[65]
Zhang	China	PD	EEG, Gait, EMG^c^	Custom (HC = 34, PD = 74)	2022	[66]
Ilias	Greece	AD	Text, Speech	ADReSS (HC = 78, AD = 78)	2023	[67]
Rallabandi	India	AD	MRI, PET	Custom (HC = 605, AD = 493)	2023	[68]
Goel	India	AD	MRI, PET	ADNI (HC = 210, MCI = 210, AD = 210)	2023	[69]
Chai	China	AD	EEG, handwriting	Custom (HC = 39, MCI = 40)	2023	[70]

^a^Dataset size records the number of participants in the study

^b^*HC* healthy control, *MCI* mild cognitive impairment, *AD* Alzheimer’s disease, *PD* Parkinson’s disease, *DLB* dementia with Lewy bodies

^c^*RBD* rapid eye movement sleep behavior disorder, *EEG* electroencephalogram, *DTI* diffusion tensor imaging, *CSF* cerebrospinal fluid, *DWI* diffusion-weighted imaging, *EMG* electromyogram

^d^A custom dataset refers to a dataset that is specifically created or collected for this particular research project or study

## Narrative synthesis of relevant findings from the evidence

### Biomedical imaging

In general, biomedical imaging data from MRI, PET and CT brain scans combined with other types of non-imaging data were effective in improving the performance of automated diagnosis of AD and PD. Several studies found that using more than one imaging modality performed better than single modal analysis [33, 42, 53]. For classifying healthy controls from those with AD, multimodal studies using MRI and PET data reported accuracy ranging from 74.3 [33] to 98% [52]. For discriminating MCI, AD and HC, the accuracy was generally lower with accuracies ranging from about 72–86% [52, 55]. For classifying PD and HC with combined MRI and PET scan data, accuracies ranged from 88.57 [44] to 98.17% [31]. The most common multimodal combinations of data were MRI plus PET (n = 10 studies), followed by MRI plus cognitive data (n = 7 studies) and MRI plus genetic data (n = 6 studies).

When multimodal imaging data was used in the included studies, the workflow of the model usually consisted of feature extraction, feature selection, feature fusion and using multi-source discriminative features for classification [53]. Convolutional neural network (CNN) was the most widely used technique for feature extraction [17, 42, 43, 53, 57, 68, 71]. After extraction of biomedical image features, feature selection was used to explore deep common features among different image features and gain information sharing among multiple modal data [53]. In the feature fusion stage, most studies used a latent feature representation space to fuse their multimodal features [17, 43]. Some also used Depth Polynomial Network (DPN) to add linear constraints on multimodal data for feature fusion [27]. In the classification stage, some studies directly used discriminative features for classification [42, 53], whereas others used additional techniques, such as hierarchical feature representation and latent representation, to enlarge the contributions of discriminative features across different modalities [17, 27, 33].

In terms of the AI methods used, a study published in 2021 reported an accuracy of 97.95% for the classification of controls and PD patients using Support vector machines (SVMs) [28]. Rallabandi et al. [68] prediction and achieved an accuracy of 98.81% on prediction of MCI-to-AD conversion in 5 years.

### Cognitive score

Cognitive scores were used in 11 studies, all related to AD [36, 37, 39, 40, 42, 50, 54, 58, 60, 62, 63]. The most common multimodal combinations of data were cognitive scores and MRI (n = 6 studies), followed by cognitive scores plus speech data (n = 5 studies) and cognitive scores plus genetic data (n = 3 studies).

In multimodal studies, cognitive scores were derived as numerical data from cognitive assessments. These scores are typically obtained by administering standardized cognitive tests that are designed to evaluate an individual’s memory, attention, language, visuospatial skills, and executive functions. Both Mini Mental Score Examination (MMSE) and more detailed neuropsychological tests assess the cognitive status of the participants. Song [40] proposed a model for discriminating early AD diagnosis and MCI and achieved accuracies of 94.44%. Rohanian et al. [50] used the cognitive (MMSE score combined with speech data to discriminate MCI from AD dementia and achieved an accuracy of 79.2% In some of the multimodal studies, cognitive data was used as the input of the deep learning model. Sanchez-Reyna et al. [39] used cognitive scores and other features as input and trained a multivariate model, achieving an Area Under the Curve (AUC) of 87.63%. Song et al. [40] developed a model called Auto-Metric graph neural network (AMGNN) and used cognitive test scores and MRI data as inputs to calculate the importance of the modality in the weight matrix. This work achieved an accuracy of 87.50% between sMCI (single-domain MCI) and pMCI (amnestic MCI with impairment in multiple domains).

### Speech and language

Speech and language deficits are recognized as predictable features in the early diagnosis of AD and PD [72]. Most multimodal studies focused on natural language processing (NLP) and related machine-learning techniques. In the speech and language multimodal studies, audio and text features were often extracted by Long Short-Term Memory (LSTM) models [37, 38, 50, 54], which refers to aligning audio and text data in time so that the machine learning model can analyze the relationship between spoken and written words. The most common multimodal combinations of data were speech plus hand movement features (n = 6 studies), followed by speech plus text (n = 5 studies) and speech plus cognitive scores (n = 5 studies). Most studies have shown encouraging outcomes when utilizing machine learning to discriminate AD from HC by analyzing speech and language characteristics, with accuracy rates ranging from 78.7% [36] to 97.3% [30]. Only two studies examined speech (with clinical data and handwriting data) for discriminating PD from HC and found a classification accuracy of 98.8% [31] and 97.62% [34].

In terms of the AI methods used, Martinc et al. [37] employed an Active Data Representation (ADR) technique for voice processing as a framework for fusion of acoustic and textual features at the sentence and word level. Nasreen [46] examined the role and contribution of interactional features in dialogue to predict whether a participant had AD; they achieved 83% accuracy using dysfluency features, 83% accuracy using interactional features, and 90% accuracy when combining both feature datasets. Pandey et al. [41] proposed a multimodal fusion-based framework that uses both speech and text transcripts to detect AD. They obtained an accuracy of 81% between AD and HC participants using a simpler architecture, reduced computational load, and complexity. To increase the effectiveness of the classification, Ying et al. [59] used fine-tuned Wav2Vec2.0 model and deep linguistic features extracted using fine-tuned Bidirectional Encoder Representations from Transformers (BERT), to classify AD patients with a support vector machine classifier and achieved 89.1% classification rate for health control vs AD. Ilias et al. [67] introduced a novel method using the Vision Transformer and cross-modal attention layers to detect dementia from speech and language modality. The results indicated that the Vision Transformer outperformed other models, and the proposed method achieved an accuracy of 88.83% for AD vs HC classification.

### Movement data

Motor function is known to decline throughout in both AD and PD. Analysis of gait, hand and eye movements is readily available in clinics using simple movement sensors [73]. There are 4 studies related to AD and 7 studies related to PD. The most common combination of data was movement data and speech (n = 5 studies) with one study focusing on AD [47] and 4 on PD [29–31, 34], followed by movement data and EEG signals (n = 3 studies, with 1 study in AD and 2 in PD) [26, 55, 66].

#### Gait

Two AD related studies [47, 55] and 4 PD [29, 30, 32, 66] studies used gait (walking) movement data. Generally, gait data was collected whilst participants walked at their usual pace over nine meters with a marker-based motion capture system (camera or sensor-based). This data was then extracted and filtered to other numerical movement features including gait speed, step/stride length, rhythm (e.g., step/stride time), variability (e.g., step/stride time variability), left-right asymmetry (e.g., the difference between left-right step/stride time), and postural control (e.g., maximum toe clearance) etc [47].

For classification of AD and MCI from HC, the gait data was often combined with speech and drawing modalities with accuracies ranging from 0.73 to 0.93 [47, 55]. Yamada [47] focused on AD and MCI and used gait, speech, and drawing behaviours to classify patients from MCI and HC. The study found that combining all three modalities led to superior classification accuracy (0.93 for AD vs controls, and 0.93 for MCI vs controls) compared to using individual modalities (0.81).

For classification of PD from HC, gait data was often combined with speech, MRI and EEG modalities with accuracies ranging from 0.85 to 0.97 [29, 30]. Garcia et al. [29] used i-vectors extracted from speech, handwriting, and gait data to classify PD patients and HC and achieved an AUC of 0.85. Two fusion strategies were tested: concatenating the i-vectors to form a super-i-vector with information from all three modalities, and score pooling. The study found that the super-i-vector fusion method led to better classification results compared to separate analysis with each modality. Vasquez et al. [30] suggested the need for further experiments with more tasks to validate the language independence of the approach and used CNNs trained with time-frequency representations (TFRs) allow for interpretation of the hidden representations of the neural network. The proposed method accurately classified PD patients and HC with an AUC of 0.97.

#### Eye movements

Eye movements are known to change in both AD and PD and data can be collected in clinic such as fixations, saccades, and pusuit movement parameters. Fixations refer to when a person’s gaze stays in one place for 60ms or longer, and saccades refer to quick movements between fixations, whereas pursuit describes tracking movements [51]. Eye movement data has been used with a wide range of modalities, including EEG, olfactory loss, handwriting and speed in one study of AD [51] and 2 PD studies [26, 34].

For AD related studies, Jang et al. [51] used a deep learning method and fused the eye movement and language modalities, yielding an overall AUC of 0.83 for AD and MCI classification. In PD related studies, Prashanth et al. utilised non-motor data such as sleep EEG (recording Rapid Eye Movement (REM) sleep behaviour disorder) and olfactory (sense of smell) data, along with other biomarkers, to classify early PD subjects from HCs using machine learning algorithms; the results show that the Support Vector Machine (SVM) classifier had an AUC of 0.964 [26].

#### Hand movements

Research has shown that changes in hand movements can occur in individuals with AD and PD and this can be used as a tool for early detection [30, 31, 46, 74]. Several studies combined handwriting and/or drawing with other modalities such as gait and speech data [75]. There was one AD study and 4 PD studies using hand movements as part of a multimodal feature analysis for the classification of neurodegenerative disorders from HCs.

For AD related studies, researchers investigated whether combining data from three modalities (hand movement, gait and speech) could improve the accuracy of AD and MCI diagnoses compared to using individual modalities alone. The study found that combining data from all three modalities achieved 89.5% accuracy for classifying AD, MCI, and HC participants, while using gait and speech modalities achieved 88.6% [46].

In PD, Taleb et al. used handwriting and speech modalities for PD classification and achieved 97.62% accuracy for discriminating early PD from controls. They proposed a combination of CNN-BLSTM (Convolutional Neural Network-Bidirectional Long Short-Term Memory) models trained with jittering and synthetic data augmentation approaches [34]. Another study fused three modalities (handwriting, speech and gait) to classify PD and HC subjects with an accuracy of 97.3%. The study also suggested that the proposed approach can be extended to other applications such as detecting prodromal stages of the disease [31].

### Others

Other types of data has also been shown effective to aid the discrimination of AD and PD from controls, such as genetic data [76], and other numerical data related to electroencephalograms (EEG; a measure of brain electrical activity), sleep and olfactory function and body fluid biomarkers.

#### Genetic data

The presence of a range of genetic mutations can indicate an increased risk of developing AD or PD. Compared to traditional studies of AD and PD that rely solely on single neural imaging data or speech data, the use of genetic data in a multimodal approach has been shown to result in better classification performance. There were 5 studies using genetic data related to AD [33, 40, 52, 56, 63] and 2 to PD [28, 44]. Genetic data were extracted as normalized numerical data from 0 to 1 indicating the risk [39] and the most common multimodal combinations were genetic data plus MRI data (n = 6 studies).

In AD, Sheng et al. [52] used genetic data to aid prediction of AD and MCI and reduce the dimensionality of the features and to address the large differences in feature scales between genetic and brain imaging data. They then used a multimodal multi-task feature selection approach to select a set of interrelated features of brain imaging phenotypes and genetic factors. By combining imaging and genetic data, the method achieved an average classification accuracy of 98% for HC and AD, 82% for HC and Early-MCI, 86% for HC and Late-MCI. Zhou et al. [33] proposed a novel latent representation learning method that used genetic data along with other modalities such as MRI and PET scans to learn a common latent feature representation and modality-specific latent feature representation. After adding genetic data, the AUC improved from 0.716 to 0.755 for discriminating stable MCI (did not convert to AD) from progressive MCI (converted to AD within 36 months).

In PD, Bi et al. [44] proposed a novel model called clustering evolutionary random neural network ensemble (CERNNE). The CERNNE was applied to form a multi-task analysis framework that discriminated PD patients and predicted PD-associated brain regions and genes. The use of genetic data allowed the CERNNE to detect altered fusion features of patients with PD, which contributes to the classification of PD from HC with an AUC = 0.88.

#### Brain electrical activity

Electroencephalogram (EEG) signals can assist in the diagnosis of neurodegenerative disorders by providing information on the neural activity of the brain. There were 3 AD studies [55, 60, 70] and 1 for PD [66] related to EEG data. EEG signals are collected using a cap-type electrode device placed on the participant’s head. The electrodes measure the electrical activity produced by the brain and transmit the signal to a recording device. EEG microstates, specifically using transition probabilities and a newly defined time-factor transition probabilities feature, can measure the severity of Alzheimer’s disease (AD) and mild cognitive impairment (MCI), distinguish between AD and MCI, and serve as a neurobiological marker for AD [77].

In AD studies, when EEG and gait parameter data were combined, Min et al. improved the ability to discriminate individuals with MCI from HCs from an AUC of 0.6711 with gait data to an AUC of 0.7267 with EEG data combined [55]. Moreover, Chai et al. [70] proposed an automated, non-invasive detection protocol for MCI based on handwriting kinetics and quantitative EEG analysis. The study used a classification model based on a dual fusion of feature and decision layers and achieved a classification result of 96.3% for MCI vs HC by using SVM with RBF kernel as the base classifier.

In PD studies, Zhang et al. [66] implemented a protocol to detect ‘freezing of gait’ (FOG, a feature of walking in PD) to classify PD participants from healthy controls with an accuracy of 0.93.

#### Sleep behaviours and olfactory loss

Studies have indicated that there are changes in the sleep-wake cycle, and in sleep behaviours, in both AD and PD. Olfactory (sense of smell) loss is also commonly observed in individuals with AD and PD. Prashanth et al. were able to classify participants into PD and HC groups using sleep behaviour and olfactory function data with an accuracy of 96.40 % [26].

#### Cerebroespinal fluid (CSF)

Cerebrospinal Fluid (CSF) is a clear, colourless liquid that surrounds the brain and spinal cord, and contains various substances that can assist in the diagnosis of brain disorders. For example, the presence of certain proteins in the CSF, such as amyloid beta and tau, can indicate the presence of AD. CSF tests are invasive (requiring a spinal needle to be passed under local anaesthetic into the lower spine) but clinically accessible for neurologists, and have been used for many decades for investigating a range of brain disorders. Dong [43] used a high-order Laplacian regularized low-rank representation (hLRR) technique to handle the noisy and heterogeneous multimodal data from CSF and clinical data and achieved an 85.32% classification accuracy between MCI and HC. However, it was reported that this method requires relatively higher computation cost of using sparse representation technique to construct hypergraphs.

### Discussion and limitation

This scoping review identified 46 papers comprising 11,750 participants, with 3569 AD, 978 with PD, 4523 with MCI and 2482 healthy controls. It demonstrated that multimodal analysis of clinically accessible data for early detection of AD and PD is a relatively new approach that has largely only emerged over the last 5 years, with 40 of the 46 articles (86.95%) published from 2019 onwards. For discriminating AD from healthy controls, a combination of MRI and PET scans with cognitive scores have been found to be highly effective with a classification rate of 98%. For AD vs MCI, a combination of MRI and PET scans along with cognitive scores achieved classification accuracies of 86%. For discriminating PD from healthy controls, the most effective combinations of data were gait, handwriting/drawing and speech data with reported accuracies ranging from 90 to 98%.

In general, MRI and PET brain imaging data was effective in improving the performance of automated detection of AD and PD. Most multimodal studies focused on computer vision (CV), natural language processing (NLP) and related machine-learning techniques. Gait, hand, and eye movement data have also been shown to assist in the diagnosis of AD and PD, with gait data being used in five studies.

In terms of the AI techniques applied to multimodal datasets for the early detection of AD and PD, the workflow of most models usually consisted of feature extraction, feature selection, feature fusion, and using multi-source discriminative features for classification. Convolutional neural network (CNN) was the most widely used technique for feature extraction. After extraction of biomedical image features, feature selection was used to explore deep common features among different image features and gain information sharing among multiple modal data. In the feature fusion stage, most studies used a latent feature representation space to fuse their multimodal features. In the classification stage, some studies directly used discriminative features for classification, whereas others used additional techniques to enlarge the contributions of discriminative features across different modalities. The accuracy rates of using multimodal features to detect AD ranged from 74.3 to 97.95%, and to detect PD ranged from 78.7% to 98.8%. The accuracy rates of using multimodal features to discriminate MCI (earlier stage AD) from AD dementia ranged from 72.67 to 88.57%. The accuracy of deep learning models for detecting AD and PD varied depending on various factors, such as the size and quality of the dataset, the complexity of the model architecture, and the specific diagnostic task.

The strengths of this study are the widespread search of data over the last 10 years, our interdisciplinary team approach bringing together the expertise of computer scientists and a clinician specializing in neurodegenerative disorders, robust methodologies following PRSISMA and the summary of evidence based both on outcomes as well as AI approaches used.

However, it is important to also acknowledge the limitations. We note that it was challenging to compare results from these studies as there were variations in terms of sample sizes with some quite small (n $\le$ 200 participants in 29 (63%) studies, and differences in study design. Moreover, the majority of applications were limited to extracting biomarker information using different networks and then using a statistical model to classify HC, MCI and AD or PD, this type of research is limited by the inability to fuse distinctive features together and the lack of relationship analysis between biomarkers. During our scoping review of 46 papers, we discovered that none of them had integrated all four types of multimodal data (images, speech, cognitive, and movement data) into a single classification model. Due to the complexity of AD or PD diagnosis, most of the experiments were done in the clinical setting, which makes it particularly difficult to construct a large multimodal dataset for the early detection of these disorders.

Moreover, the challenge with integrating multimodal data is that they are often incomplete, contain noise information caused by different data collection tools (e.g. different MRI scanners) or protocols (e.g. gait assessment for 1 min vs 10 min, or for maximal speed vs comfortable paced walking), and have missing data. To overcome these challenges, researchers have proposed novel methods for high-order Laplacian regularized low-rank representation, latent representation learning, and dataset enrichment. Existing multimodal methods have mainly focused on classifying cognitive status using different neuroimages (MRI and PET) with other non-imaging variables [28, 32, 33, 35, 42, 44, 52, 53]. However, collecting neuroimaging data such as MRI and PET scans are expensive, and not ideal for a large population.

In this scoping review, we aimed to summarise the evidence for multimodal methods that would inform the development of low-cost, reliable tests for the early detection of AD and PD in clinical settings. Meanwhile, detecting diagnostic biomarkers that are non-invasive and cost-effective is of great value not only for clinical assessments but also for epidemiological studies (that may require home tests) and research purposes. Further research is needed to validate the findings and determine the effectiveness of multimodal learning in aiding diagnosis of AD and PD.

## Conclusion

In conclusion, our scoping review and study evaluated the multimodal analysis of clinically accessible data for early detection of AD and PD, the two most common neurodegenerative disorders. The multimodal learning analysis is a relatively new approach, with 86.95% (40/46) studies published in the last 5 years (and 69.57% (32/46) studies in the last 2 years), employs data from biomedical imaging, cognitive, speech and language, gait, hand, and eye movement tests, along with EEG and genetic assessments.

The studies highlight that the classification rates using multimodal data are promisingly high, not only in distinguishing AD and PD from healthy controls but also differentiating between AD and MC. The crucial role of MRI and PET brain imaging data in enhancing automated detection has been underscored, with Convolutional Neural Networks (CNN) frequently employed for feature extraction. However, existing multimodal methodologies primarily focus on classifying cognitive status using brain scan image data and non-imaging variables, which, while effective, are costly and impractical for population-level tests.

Despite the substantial progress, several challenges need to be addressed. Comparing outcomes across studies is difficult due to variations in sample sizes, study designs, and limitations of datasets. Furthermore, despite extensive utilisation of multimodal data, none of the studies integrated all data types into a single classification model, marking a critical area for future research. Additionally, handling issues of incomplete or noisy data calls for more advanced techniques such as high-order Laplacian regularized low-rank representation and latent representation learning.

With the escalating prevalence of AD and PD, our findings call for more rigorous research, not only to validate the current results but also to discover low-cost, reliable, and non-invasive methods for early detection in both clinical and remote home settings. The overarching goal is to integrate different types of multimodal data to develop accurate models, thereby contributing to better patient care, facilitating new drug development, and advancing the promising trajectory of multimodal AI in the realm of neurodegenerative disorders.
